# High-Capacity Optical Fingerprinting Using Dual-Peak Photoluminescence of Quantum Dots

**DOI:** 10.1021/acsami.5c19508

**Published:** 2025-12-22

**Authors:** Syeda Ramsha Ali, Stephen V. Kershaw, Yinglong Zhu, Ahmed A. Z. Dawoud, Yueyu Guo, Kees De Groot, Nema M. Abdelazim

**Affiliations:** † School of Electronics and Computer Science, 7423 University of Southampton, Southampton SO17 1BJ, U.K.; ‡ Department of Physics and Materials Science, 53025 City University of Hong Kong, Kowloon 999077, Hong Kong, China; § Microelectronics Thrust, The Hong Kong University of Science and Technology (Guangzhou), Guangzhou 511455, China; ∥ School of Biological Sciences, 7423 University of Southampton, Southampton SO17 1BJ, U.K.

**Keywords:** optical PUFs, quantum dots, security, nanomaterials, photoluminescence, Hamming distance, encoding capacity

## Abstract

Counterfeiting and unauthorized duplication continue to pose significant threats across industries, ranging from electronics to pharmaceuticals. In response to this challenge, we present a novel optical fingerprinting platform based on cadmium-free CuInS_2_/ZnS quantum dots (QDs), which exhibit a distinctive dual-peak photoluminescence (PL) signature. Time-resolved PL (TRPL) analysis confirms the distinct recombination origins of the two peaks, supporting the assignment to core- and interfacial/shell-related states. Our approach extracts two intrinsically coupled emissions from a single QD type, where both peaks originate within the same nanostructure, making the fingerprint inherently unclonable. This phenomenon enables the generation of rich tunable spectral profiles across a selected range of excitation wavelengths. Using spectral-to-digital processing, we extracted three features from both emission peaks under 10 excitation wavelengths to generate binary fingerprints. The resulting theoretical encoding capacity is estimated to be 1.2 × 10^18^ compared to an experimental error probability of ∼3 × 10^–17^. These findings validate the strength and security of the proposed fingerprinting system, highlighting its practical potential for anticounterfeiting applications.

## Graphical abstract

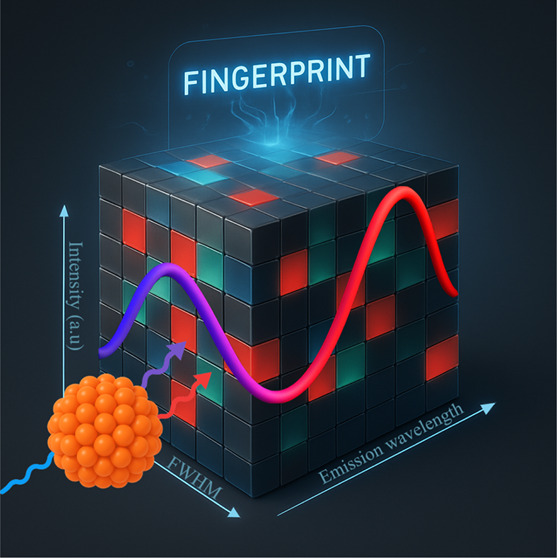

## Introduction

With the continuous development of technology and the growth of global trade, the problem of counterfeit and inferior products has become increasingly serious. In 2016, the international trade in counterfeit and pirated goods was estimated to reach $509 billion, accounting for approximately 3.3% of global trade.^1^ Counterfeit products not only result in substantial economic losses but also pose serious threats to public health and safety, particularly in the areas of pharmaceuticals and food.^2^ Traditional anticounterfeiting techniques, such as barcodes, holograms, and watermarks, rely on visible patterns and often suffer from limited complexity. They are vulnerable to tampering and increasingly susceptible to replication due to the availability of advanced fabrication tools.^3^ To address these challenges, optically unclonable functions (PUFs) have emerged as a promising next-generation solution. Unlike traditional methods, optical PUFs influence the inherent and uncontrollable physical variations that arise naturally during the fabrication of the materials. These random physical variations serve as the foundation of a challenge response pairs (CRPs): a challenge, which is a random or predefined input (stimulus), and a response, which is the corresponding output (reaction) generated by a system. CRPs are used in an authentication protocol for optical PUFs, where each challenge is processed by a unique physical structure to produce a specific response. The uniqueness and unpredictability of CRPs make them valuable for secure identification, anticounterfeiting, and cryptographic key generation. These physically derived “fingerprints” make optical PUFs highly resistant to cloning and tampering, positioning them as a powerful tool for secure authentication and advanced anticounterfeiting applications. Among the various material platforms explored for optical PUFs, fluorescent materials have become increasingly prominent in anticounterfeiting applications due to their high sensitivity and ability to be easily incorporated into various substrates, providing enhanced security against forgery.^4^ Material technologies in this area include organic dyes,^5^ carbon dots,^6^ upconverting nanoparticles,^7^ luminescent materials,^8^ particles,^9,10^ and wrinkles/crystals.^11^ Data encryption techniques need constant improvements to remain up-to-date as the world progresses. Many encryption techniques that use fluorescent materials rely on simple fluorescent colors as coding elements, but this approach is no longer sufficient to meet modern data encryption requirements.^12^ Also, fluorescent materials often rely on single-color emissions, limiting their effectiveness in advanced security systems. Single-color systems are susceptible to explicability under visible light, making them less viable for high-level encryption. To overcome these limitations, research efforts have focused on designing multicolor systems capable of emitting in red, green, and blue (RGB) regions.^13,14^ Such systems enable dynamic and multimodal optical information encoding, allowing for enhanced anticounterfeiting measures.^15^ A key challenge in developing multicolored systems lies in achieving consistent excitation across all colors while ensuring strength and stability for real-world applications.

Quantum dots (QDs) have emerged as a groundbreaking choice for optical PUFs due to their unique combination of tunable optical properties, quantum confinement effects, and nonlinear optical characteristics.^16^ These nanoscale semiconductor particles exhibit size-dependent photoluminescence (PL), allowing precise control over emission characteristics.^17^ This inherent tunability not only provides a rich source of CRPs but also makes QDs versatile for applications in secure information storage and anticounterfeiting.^14,18^ The QDs can generate dual or multipeak emissions,^19,20^ using both band-edge transitions^21^ and defect states,^22^ which enhances the randomness and number of CRPs.^23^ Their exceptional brightness and quantum efficiency ensure strong signal integrity, while their compatibility with various matrices, such as polymers or solvents, facilitates diverse practical implementations.^24^ Furthermore, QDs’ unique electronic and optical properties, along with their potential for integration and surface modification, make them ideal for their use in cryptographic applications.^25^ QD-based optical PUFs use their unique PL properties to generate highly complex and multidimensional optical responses from a single pixel, substantially increasing the encoding capacity of the system. Encoding capacity is the total number of unique CRPs that a system can generate.^26,27^ The nanoscale emission properties and spectral multiplexing capabilities of QDs allow for a vast number of CRPs, enabling the generation of unique and irreproducible optical signatures. The ability to encode multidimensional optical responses, such as intensity variations, polarization or phase changes, further strengthens the resistance of these optical PUFs against physical and computational attacks.^28^ Additionally, QDs-based optical PUFs exhibit compatibility with advanced photonic architectures, facilitating seamless integration into secure authentication systems across diverse applications.^29^ While environmental stability is a critical consideration for optical PUFs, QDs-based systems can be effectively engineered with tailored surface passivation and encapsulation strategies to ensure long-term durability and performance.^30^ During the synthesis of QDs, strong surface effects arising from their large surface area can promote close interactions among nanoparticles. Polymers are used for surface passivation and to maintain the nanoscopic integrity, and by mixing polymers into QDs, the stability and optical properties of the QDs can be improved.^31^ Poly­(methyl methacrylate) (PMMA) is widely used as a polymer host for QDs owing to its excellent mechanical strength, optical transparency, and ability to encapsulate nanomaterials against oxygen, moisture, and thermal fluctuations.^32,33^ The dynamic photophysical properties of QDs, such as their excitation-dependent emission, introduce an additional layer of security, making these optical PUFs increasingly resistant to forgery. The integration of these properties with computational tools enables the seamless translation of material-specific randomness into digital cryptographic frameworks.

In this work, we explore the potential of dual-emission behavior in QDs systems as a foundation for optical fingerprinting. By the use of two well-resolved PL emission peaks as independent features, a multidimensional framework for physical identity encoding is constructed. Rather than relying on complex materials or fabrication steps, our approach emphasizes simplicity, repeatability, and scalability. We employed a simple spin-coating process for QDs deposition, enabling compatibility with a range of substrates, while encapsulation in a polymer matrix provided enhanced QDs stability.^30,31^ The spectral information embedded in these dual peaks, captured under varying excitation stimuli, is transformed into structured digital representations that serve as unique identifiers. Through systematic analysis of their stability, encoding potential, and reproducibility, we demonstrate the effectiveness of this dual-peak PL-based strategy as a reliable and high-capacity platform for optical security and authentication applications.

## Results and Discussion

Figure 1 illustrates the conceptual framework and comparative encoding behavior of two classes of QD systems: cadmium selenide/zinc sulfide (CdSe/ZnS) and copper indium sulfide/zinc sulfide CuInS_2_/ZnS (CIS). The process begins with the preparation of QDs, Figure 1a, where the QDs are deposited through a spin coating technique. Figure S1 presents the Atomic Force Microscopy (AFM) characterization used to determine the film thickness of the deposited film. The AFM height image in Figure S1a with the extracted line profile in Figure S1b clearly shows a distinct step edge, and film thickness is approximately 108 ± 3 nm, representing the average thickness of the deposited layer. The AFM analysis revealed an average surface roughness of ∼22.6 nm, indicating a nonuniform surface morphology. Such inherent randomness in the film structure is beneficial for our fingerprint generation, where a random optical response from the deposited layer is desired. These QDs were either dispersed in solution without PMMA or embedded in a PMMA matrix. Here, PMMA not only acts as a matrix material for film formation but also provides as an encapsulating medium, enhancing the stability of the QDs.^30^ As highlighted in Figure 1b, the approach offers several key advantages, such as supporting advanced optical security through rich PL emission features, i.e., the dual peaks at varied excitation wavelengths, which allows high-capacity data encoding via feature-based digitization. It can seamlessly integrate into existing substrates, providing high encoding capacity due to spectral variability, and is based on environmental friendly materials. This last point is particularly important as QDs such as CdSe and PbS are known to raise regulatory concerns due to their heavy metal content.^34^ In contrast, CIS-based QDs are cadmium- and lead-free, exhibiting strong PL emission and chemical stability, which aligns with current eco-friendly standards.

**Figure 1:**
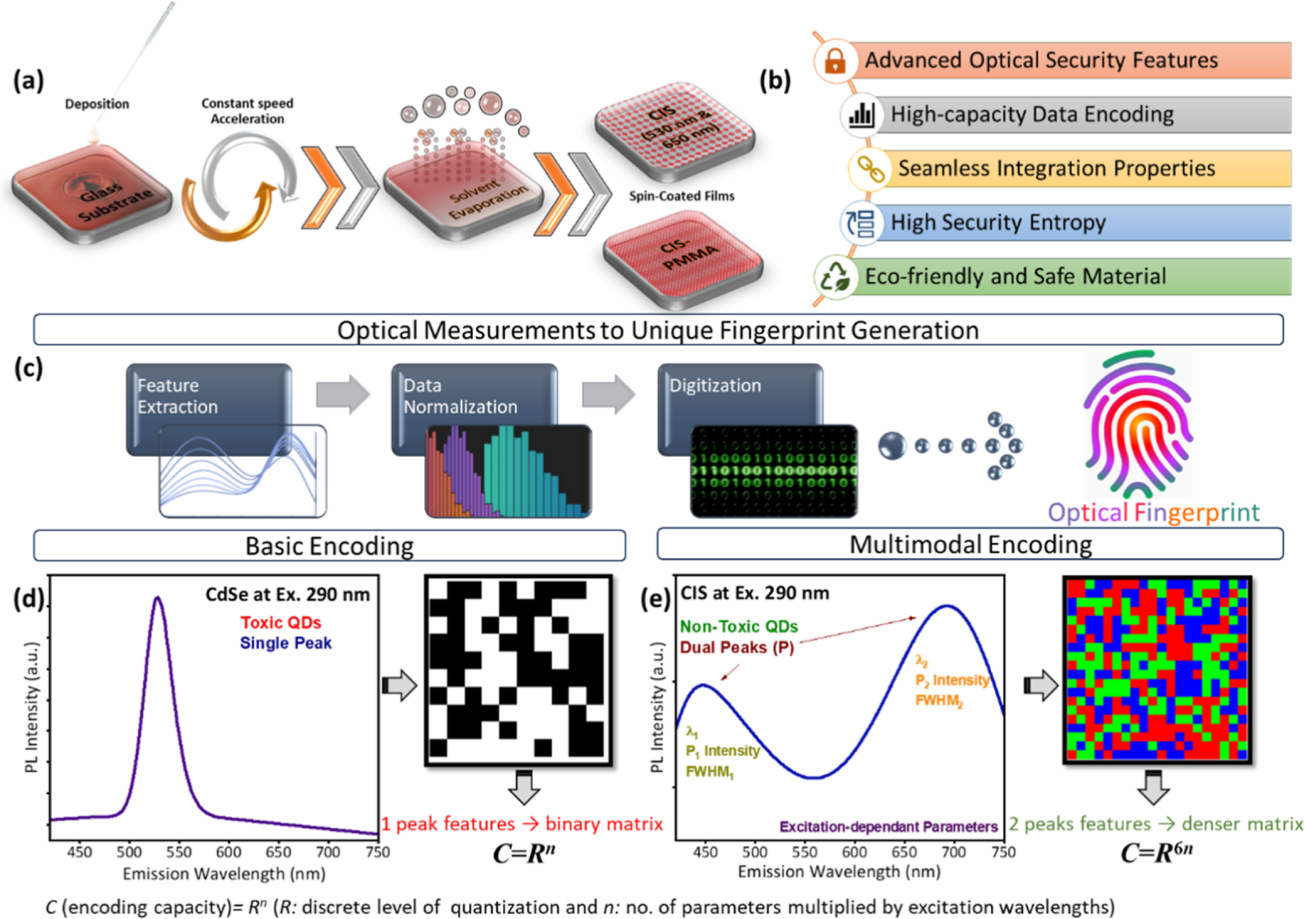
Schematic representation of the QDs-based optical fingerprinting platform: (a) spin-coating process for preparing the film composites on glass substrates, (b) core advantages of the proposed system, (c) optical workflow from PL emission acquisition to feature extraction, normalization, and digitization into digital fingerprinting codes, (d) basic encoding using CdSe/ZnS QDs with single-peak emission under 290 nm excitation, yielding limited features and lower entropy, and (e) multimodal encoding using CIS at 290 nm utilizing dual-emission behavior to extract a richer features set, enabling denser, more secure fingerprint matrices with substantially higher encoding capacity (*C*).

Figure 1c presents the digital transformation structure, where the spectral data obtained from PL emission measurements are processed through peak fitting to extract three key parameters per peak: peak intensity, peak emission wavelength, and full width at half-maximum (FWHM). After normalization, these features are digitized into binary or multilevel codes, producing compact digital fingerprints that are unique to each sample. These fingerprints form the basis for secure optical identification and are suitable for use in optical PUFs architectures. Finally, panels (d) and (e) in Figure 1 contrast two encoding regimes under identical measurement conditions. In Figure 1d, CdSe/ZnS QDs were excited at 290 nm, revealing a single emission peak. Feature extraction from this single peak provides a limited parameter space, resulting in a narrow feature set and lower encoding capacity (*C*), mathematically represented as *C* = *R* ^*n*^, where *R* is the digitization resolution and n is the total number of features.^9^ The resulting fingerprint matrix is binary and limited in complexity, rendering it potentially more susceptible to cloning or spoofing. In contrast, Figure 1e demonstrates the behavior of CIS QDs excited at 290 nm, exhibiting dual-peak PL emissions.^21,35,36^ To highlight the fundamental differences in optical behavior between the two QDs, the PL emission wavelength full range spectra for CdSe/ZnS are presented in Figure S2. It can be noted that they exhibit a sharp singular emission peak with a negligible wavelength shift or broadening, resulting in a limited number of extractable spectral features. In contrast, the CIS QDs show a characteristic dual-peak emission profile with distinct spectral responses that vary dynamically with excitation wavelength, which are discussed in detail afterward. This dual-peak architecture enables the extraction of twice the number of parameters at each excitation, thus substantially increasing the available features for fingerprint generation. As a result, the CIS-based system supports a significantly higher encoding capacity than the CdSe/ZnS system.

The presence of two well-defined peaks allows the extraction of six distinct features per excitation wavelength, significantly enriching the encoding capacity. This encoding capacity enables a more secure system that is inherently harder to clone or reverse engineer. The multicolor matrix visually conveys this increase in information density and uniqueness. The distinction between basic encoding and multimodal encoding in this context is a matter not only of emission complexity but also of functional performance. The dual-peak strategy does not require additional material layers, surface patterning, or any external modulation; the added capacity arises purely from the intrinsic photophysics of the QDs. This positions CIS QDs as a promising material platform for next-generation data encryption and optical PUFs applications where reliability, reproducibility, and eco-compatibility are equally important.

### PL Emission Characteristics

The PL emission spectra of CIS(530) and CIS(530)-PMMA, under excitation wavelengths ranging from 250 to 330 nm (in 5 nm steps), are presented in Figure 2. At shorter excitation wavelengths (250–280 nm), Figure 2a, a single emission peak, hereafter referred to as Peak I, is observed around 530 nm, originating from band-edge transitions within the CuInS_2_ core. In this regime, the excitation energy is sufficient to directly populate core states, and no other emissions are active.^37^ Upon increasing the excitation wavelength beyond 285 nm, a second emission, denoted as Peak II, emerges at higher photon energies, producing a distinct dual-emission profile, as shown in Figure 2b. The emergence, spectral position, and relative intensity of this peak evolve systematically with excitation wavelength, indicating that it originates from a mechanism different from the primary band-edge transition of the QD’s core. The presence of Peak II at energies above the core bandgap strongly suggests that it does not arise from defect-mediated recombination, which conventionally manifests below the band-edge through transitions involving intragap states.^19,38^ Alternatively, the behavior of Peak II points toward excitation-dependent carrier dynamics, such as transitions involving surface-related states, shell-to-core energy transfer pathways, or quantum confinement effects associated with compositional inhomogeneity within the QDs. These processes are prominent under higher-energy excitation (285–330 nm), thereby giving rise to the observed secondary emission that coexists with the principal band-edge peak.

**Figure 2:**
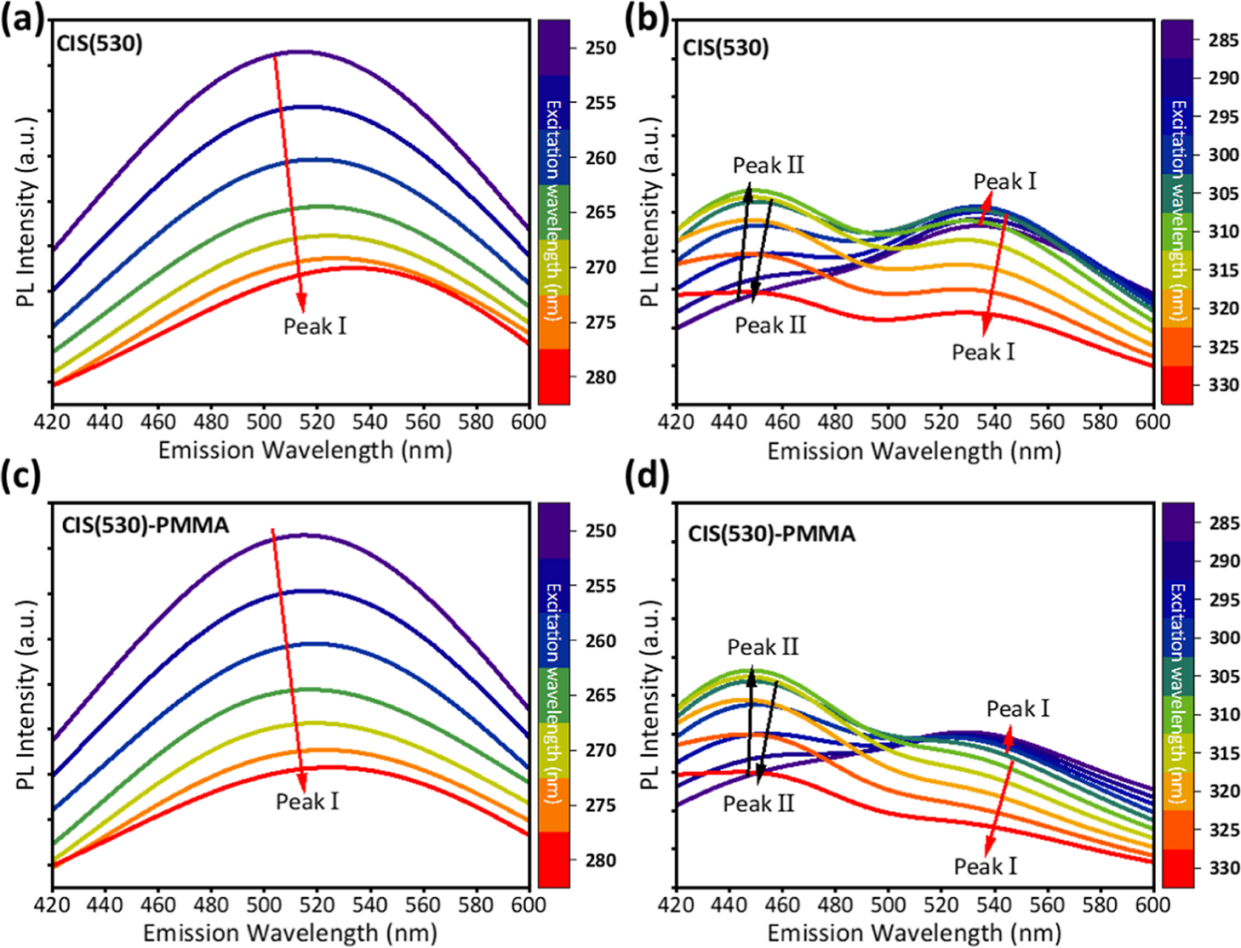
PL spectra of CIS QDs with a nominal emission wavelength of 530 nm under different excitation wavelengths. The spectra of CIS(530) (a) for the excitation wavelength range 250–280 nm and (b) for the excitation wavelength range 285–330 nm. The spectra of a CIS(530)-PMMA matrix (c) for the excitation wavelength range 250–280 nm and (d) for the excitation wavelength range 285–330 nm.

Embedding CIS(530) in a PMMA matrix, as shown in Figure 2c,d, preserves the dual-emission characteristics while improving spectral stability and suppressing background noise. The polymer matrix serves as a passivating environment that enhances emission intensity by limiting moisture and oxygen exposure.^39^ Notably, Peak II becomes more prominent at higher excitation wavelengths, which we attribute to the stabilizing effect of the PMMA matrix. PMMA provides encapsulation and supports uniform film formation, thereby protecting the QDs from environmental degradation and enabling consistent optical emission for longer times.^40^ This behavior aligns with reports of matrix-mediated modulation of local dielectric properties, which can influence shell-state recombination probabilities.^40^ The combined effect of the excitation-dependent response and matrix-assisted stabilization underpins the fingerprinting potential of these QDs, enabling dynamic tunability of the PL signature.

The CIS(650) QDs exhibit a distinct and persistent dual-emission profile, with Peak I centered at ∼650 nm and a higher-energy Peak II observed consistently across the entire excitation range from 250 to 330 nm, as shown in Figure 3a,b. In contrast, for CIS(530), Peak II appears only at longer excitation wavelengths. The presence of Peak II at all excitation conditions in CIS(650) indicates a different balance between the available emissions, likely determined by the structural and electronic configuration of the larger-core QDs. The higher-energy position of Peak II above the CIS(650) band-edge emission excludes the influence of any defect-related intragap states, which typically emit at longer wavelengths. Instead, Peak II is most reasonably assigned to recombination from shallow states located at the CIS/ZnS core-shell interface or within Zn-rich shell regions, whose emission characteristics are governed by band alignment and quantum confinement effects.^41,42^ The larger CIS(650) core and potentially thicker shells are likely to facilitate exciton delocalization across the interface. Such structural modifications have been shown in prior studies to stabilize PL, suppress emission blinking, and promote interfacial emission.^43^ The incorporation of PMMA, shown in Figure 3c,d, enhances the stability of the dual emission by providing a protective matrix that maintains the interfacial environment of the QDs. This encapsulation preserves the distinct spectral features of both peaks and prevents intensity loss and spectral distortion over time. Importantly, the intensity and position of Peak II remain relatively unaffected by polymer embedding, underscoring the strength of the underlying emission mechanism. This spectral consistency and modulation potential are critical for optical PUFs applications as they allow fingerprint generation across a broad excitation range with minimal spectral drift.

**Figure 3:**
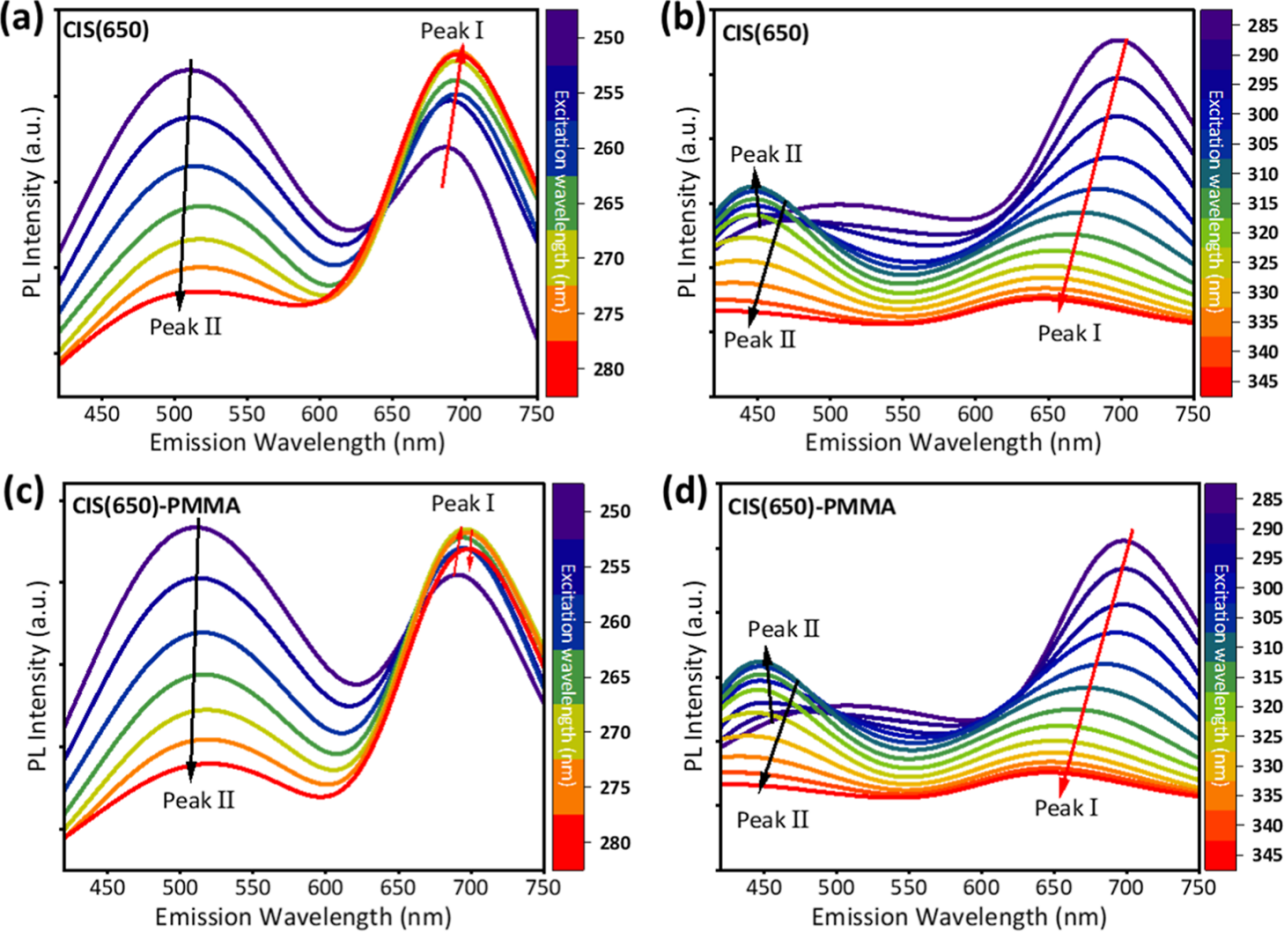
PL spectra of CIS QDs with a 650 nm emission wavelength recorded under different excitation wavelengths. (a,b) show CIS(650) spectra for excitation ranges of 250–280 nm and 285–345 nm, respectively. (c,d) The corresponding spectra of a CIS(530)-PMMA matrix for the excitation ranges of 250–280 nm and 285–345 nm, respectively.

Following the PL measurements, Gaussian fitting was applied as a phenomenological tool to isolate and quantify each peak, providing the required parameters of excitation wavelength, intensity, and FWHM. This approach enables accurate decomposition of overlapping peaks and allows for consistent feature extraction for fingerprint generation. We note that a more rigorous physical analysis of QD emission spectra would involve conversion into the energy domain (via a Jacobian transform) to account for intensity normalization per energy bandwidth. However, since the purpose of this study is not to derive a detailed photophysical model but rather to extract parameters for optical fingerprinting, fitting in the wavelength domain is sufficient and ensures consistency across all excitation conditions. The examples are shown in Figure S3, and similar fits were performed across all excitation wavelengths. The extracted parameters from the fitted graphs are shown in Figures S4 and S5. For both CIS(530) and CIS(650), Peak I exhibits relatively stable emission wavelengths (specifically for CIS(650)) across the excitation range, typically centered in the green emission window, indicative of band-edge recombination. Peak II exhibits more pronounced spectral variability, with its emission wavelength shifting depending on the excitation energy. The directions of the arrows for two peaks, in Figures 2 and 3, indicate the shift of the peaks, making the response unpredictable with respect to excitation wavelengths. This behavior is consistent with transitions sensitive to surface strain, compositional fluctuations, or trap-assisted recombination mechanisms. The intensity profiles in Figures S4 and S5 highlight this distinction, and this variability is key for encoding as it introduces additional data dimensions for fingerprint generation. Finally, the FWHM values reflect the emission quality and heterogeneity of the states involved. While Peak I maintains a relatively narrow and consistent width, suggesting well-defined monodisperse emissions,^44^ Peak II shows much greater variation in FWHM, which is consistent with interface-related transitions.^45^ These parameters form the basis for binary encoding, allowing the optical characteristics of the QDs to be translated to digital identifiers.

### Time-Resolved PL Measurements

To complement the steady-state PL data, we performed time-resolved PL (TRPL) measurements on CIS(530)-PMMA and CIS(650)-PMMA, as shown in Figure 4. The decay profiles were fitted using a biexponential model, yielding distinct lifetime components for the two emission peaks. For CIS(530), Peak I (core-related) exhibits an average lifetime of 168 ns, while Peak II (interfacial/shell-related) presents a much shorter decay of 3.6 ns. In CIS(650), the separation was even more pronounced, with Peak I reaching 357 ns compared to 6.2 ns for Peak II. These well-separated lifetimes strongly support the interpretation that Peak I arises from the CIS core, whereas Peak II originates from faster recombination pathways associated with the core-shell interface or ZnS shell.^42,46^ Notably, the faster component cannot be attributed to deep trap-mediated recombination, which typically yields long-lived decays. Such reduced lifetimes are characteristic of emission from shallow interfacial or shell states, whereas the longer-lived decay of Peak I corresponds to band-edge recombination in the CIS core.^42,46^

**Figure 4:**
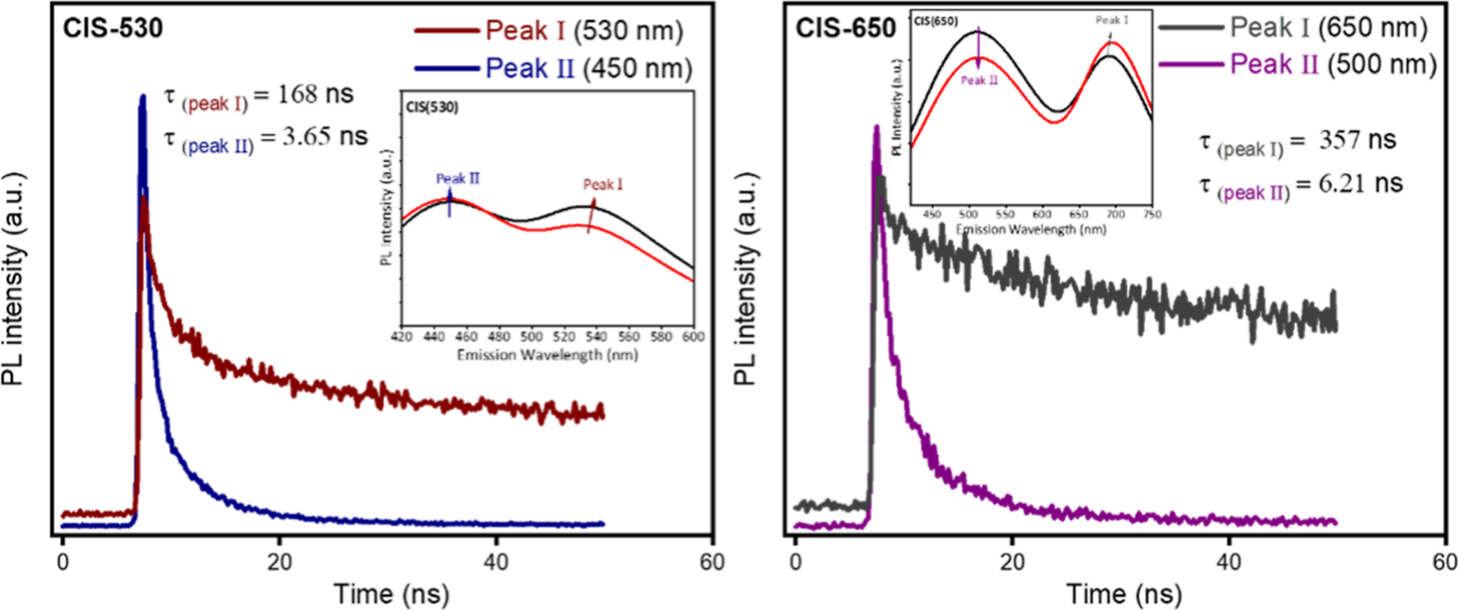
TRPL decay profiles of CIS(530)-PMMA and CIS(650)-PMMA.

Our results are therefore consistent with emission from shallow states located at the core-shell interface or within the ZnS shell, selectively accessed under specific excitation energies. Prior reports have linked such behavior to alloy composition variations, Zn enrichment, Zn-Cu-In-S phase domains, interfacial strain, and structural heterogeneity in CIS-based QDs.^40,42^ In particular, Fuhr et al.^47^ provided mechanistic insights into dual-emission behavior, demonstrating that structural heterogeneity and interfacial transitions can give rise to distinct PL emissions. Furthermore, quantum confinement within thin ZnS shells has been reported to cause blue-shifted emission that may compete with core-state transitions, depending on the excitation energy and band alignment at the core-shell interface.^40^ These TRPL results with the literature context provide direct mechanistic evidence for the dual-peak phenomenon from the core and shell of the QDs.

### Binary Encoding and Matrix Formation

The process of generating an optical fingerprint begins by encoding spectral data in a digital format. The binary data extracted from the PL emission parameters of each sample, as shown in Figures S4 and S5, are summarized in Table S1. Each peak is characterized by three key spectral parameters: wavelength, intensity, and FWHM. These parameters are binary encoded as values of 0 or 1, reflecting distinct spectral features. This encoding process transforms the physical properties of the peaks into a format suitable for mathematical operations and subsequent security encoding. The table outlines the binary encoding of the PL emission parameters for multiple samples. For each sample, binary sequences were generated from three fitted PL emission parameters obtained from two distinct peaks. The variation in bits across samples reflects the adaptive quantization based on feature scaling. Each binary segment is unique to its peak, enabling distinct digital representations for both core and composite samples. The table highlights the consistent presence of dual-peak behavior across the different material configurations and confirms that each peak contributes independently to the final digital identity of the sample. Encoding the data into binary code, instead of relying on raw parameter values, enhances compatibility with cryptographic and authentication platforms and minimizes sensitivity to noise.

Once the binary encoding is complete, the data for each peak are organized into row vectors. For Peak I, the binary values for wavelength, intensity, and FWHM are structured into row vectors A, B, and C, respectively. Similarly, for Peak II, these parameters are arranged into row vectors a, b, and c, as can be seen in step 1 of Figure 5. It illustrates the organizational logic used to structure binary information into a hierarchical encoding system. These row vectors provide a foundational structure that facilitates the next step of matrix transformations. By organizing the encoded parameters into vectors, the spectral data can be systematically processed and transformed into matrix forms. In the matrix transformation step, each row vector is subjected to a transpose-dot product operation. This process introduces structural depth by encoding the spectral data into three independent 2D matrices. The mathematical transformations are represented as (*A*)^T^ × (*a*), (*B*)^T^ × (*b*), and (*C*)^T^ × (*c*), producing three distinct matrices, namely, Matrix 1, Matrix 2, and Matrix 3 (step 2 in Figure 5). This mathematical framework encodes not only parameter-specific values but also interpeak relationships and enhances randomness and uniqueness. Each matrix represents a different parameter (wavelength, intensity, or FWHM), ensuring that the spectral characteristics are distinctly captured. The resulting matrices are then visualized by mapping the matrices to the red, green, and blue colors. Matrix 1 is used to generate the red color, Matrix 2 produces the green color, and Matrix 3 generates the blue color. This step integrates the encoded spectral data into a visual format, where each color independently represents one of the three key spectral parameters.

**Figure 5:**
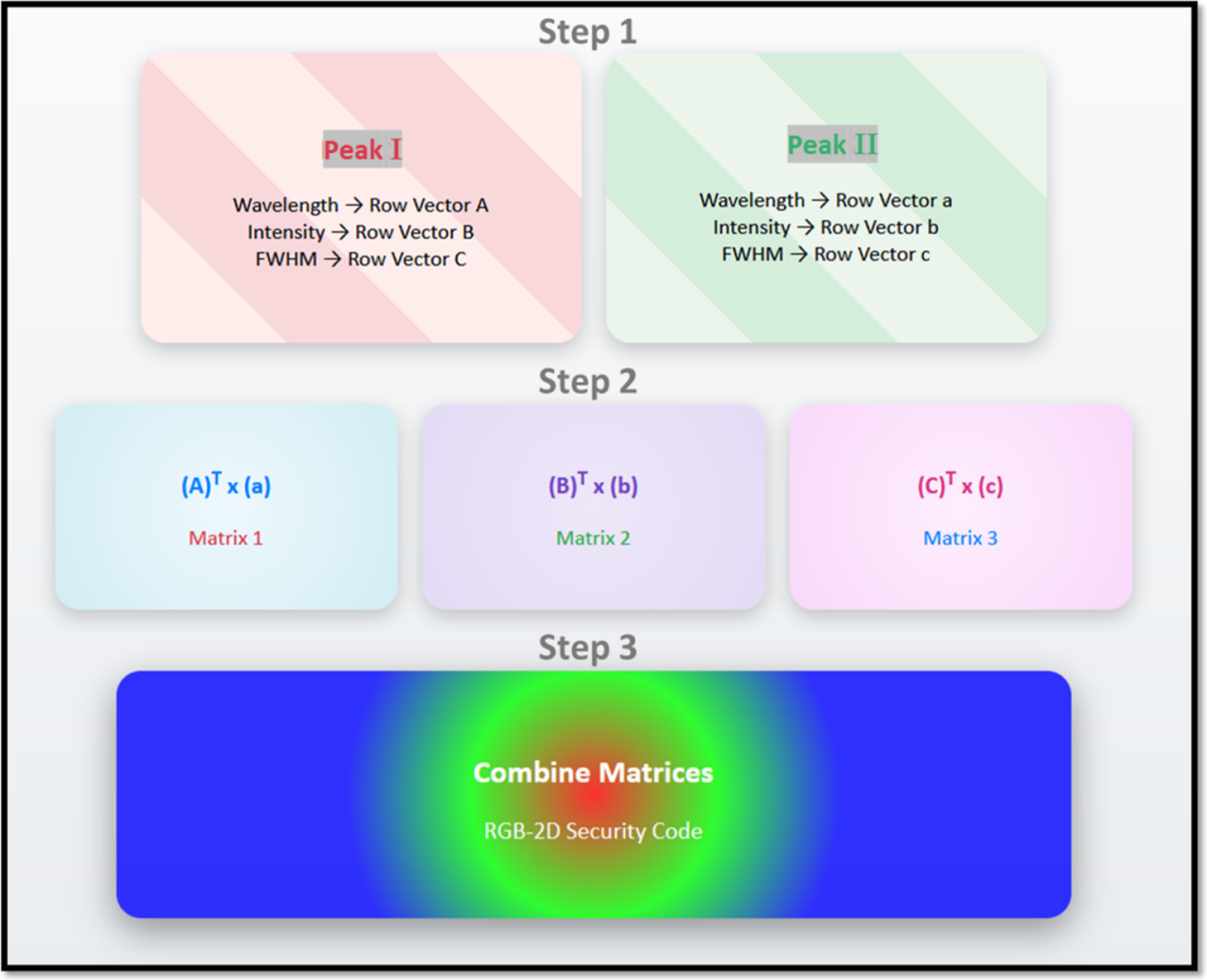
RGB matrices generated from binary-encoded PL data.

Finally, in the third step of Figure 5, the three matrices are merged to produce a composite 2D security code. This security code encapsulates the spectral characteristics of both peaks in a single multicolored output. The use of RGB not only makes the data visually distinguishable but also sets the stage for combining them into a unified security code. This security code provides a strong mechanism for encoding and visualizing spectral data, ensuring that the encoded information is both secure and verifiable. This process exemplifies the seamless combination of spectral encoding, mathematical transformations, and visual representation to produce a verifiable security code.

Additionally, as shown for CIS(530) in Figure 6a, these abstract binary matrices are translated into visually interpretable and quantified 3D data maps. This figure provides a step-by-step depiction of how binary-encoded spectral features are mapped into distinct color components. The process begins with three separate binary data matrices, each corresponding to one of the key spectral characteristics. These matrices, consisting of binary numbers, determine the spatial distribution of color in the final encoded output. The first matrix, associated with wavelength, is assigned a red color, generating a partial, red-coded pattern in the output. Similarly, the second matrix, representing intensity, is mapped to the green color, contributing to a distinct, green-coded pattern. Lastly, the third matrix, corresponding to FWHM, is transformed into a blue color representation. Once the individual matrices are converted into their respective color components, they are superimposed to generate a composite 2D output. This final encoded output integrates all three spectral parameters into a structured 2D security pattern, effectively encapsulating the unique spectral signature of the QDs. The process has been similarly followed for the rest of the samples and is presented in Figures S6a, S7a, and S8a for CIS(530)-PMMA, CIS(650), and CIS(650)-PMMA, respectively.

**Figure 6:**
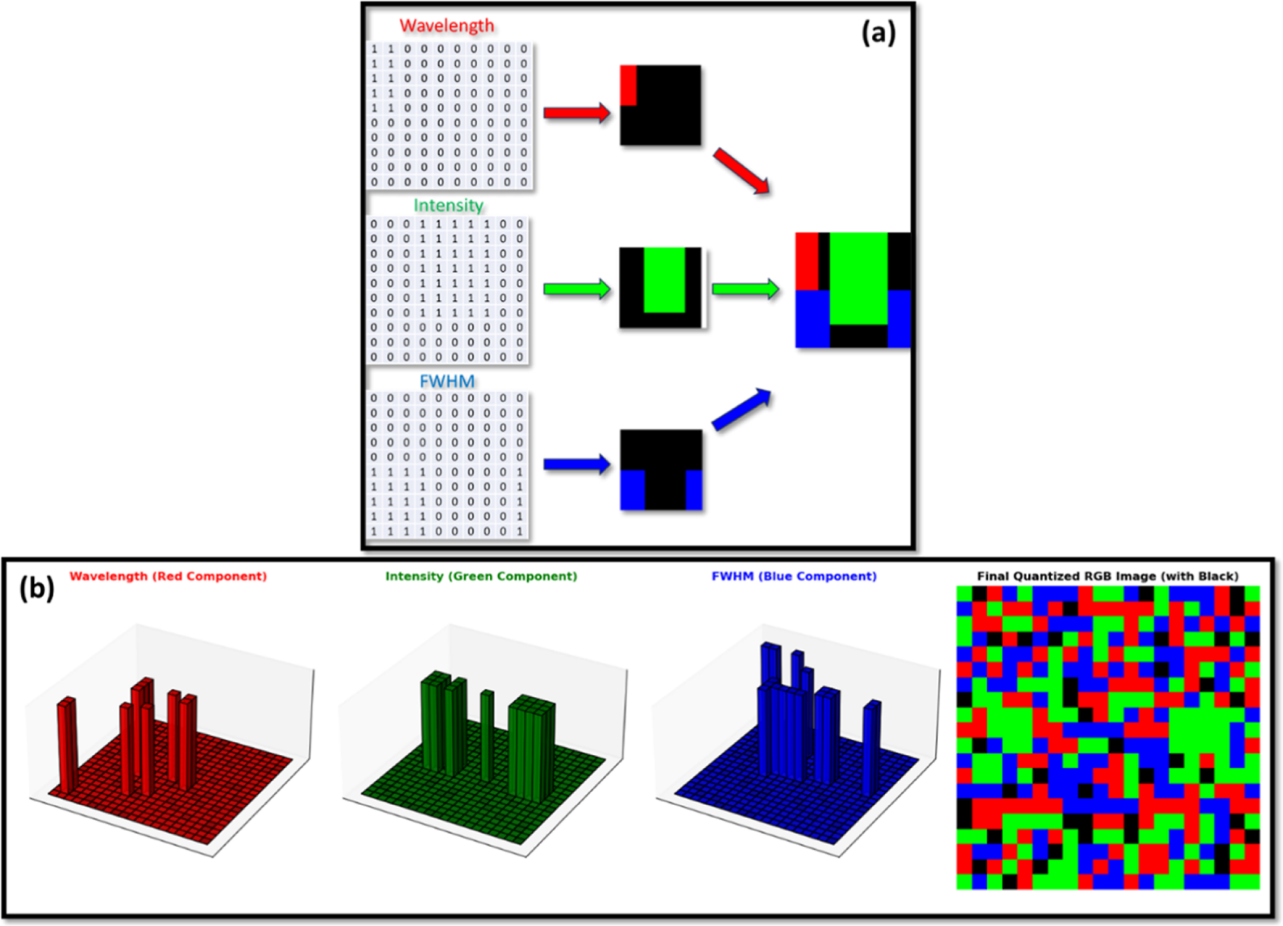
A visual representation of the process used to generate a quantized security code by encoding spectral parameters into a structured color-mapped format for CIS(530), (a) the transformation of binary spectral data into individual color components, and (b) the corresponding 3D quantization of these components, leading to the final quantized optical security display.

Figure 6b illustrates a 3D quantization approach applied to each of the three color components for CIS(530). These components are visualized as 3D bar plots, where the height, position, and number of bars represent the encoded intensity for each spatial location. This quantization step enhances the security of the 2D-colored encoded data by introducing a structured, computationally complex pattern that is difficult to replicate. Finally, all three components are merged to form the final quantized display, which converts the spectral information into a visually distinct and computationally retrievable security pattern. The quantization, performed postmerge using a Python-based mapping, ensures consistency and improves resistance to minor noise or measurement variation. This structured transformation process ensures that each optical fingerprint is unique and highly resistant to duplication, making it an excellent candidate for optical PUFs. This quantization process has been similarly followed for the other three samples and is presented in Figure S6b, S7b, and S8b for CIS(530)-PMMA, CIS(650), and CIS(650)-PMMA, respectively. Together, these three elements, the tabulated binary structure, the matrix encoding framework, and the optically quantized fingerprint, form a strong digitization framework. Unlike purely spatial or grayscale image-based optical PUFs, this system incorporates real, physics-based spectral features, structurally encoded and visually synthesized for both machine processing and human verification.

The fingerprint in our system is constructed through a three-step basis, as explained previously in detail. The three PL features extracted from each of the two PL emission peaks under multiple excitation wavelengths (Figure 5, Step 1) are stored as a vector of length *n*, corresponding to the number of excitation wavelengths. Thus, six vectors are obtained in total from Step 1. In Step 2, each of these six vectors is processed through a transformation function (matrix multiplication, dimensionality reduction) to generate intermediate matrices (Matrix 1, Matrix 2, Matrix 3) of 10 × 10 (depends on number of excitations), representing fused feature representations. In Step 3, the fingerprint is formed by combining the outputs of these matrices into a single vector. The number of features contributing to the fingerprint is 6*n* as each of the six parameters is sampled across *n* independent conditions, in this case, the excitation wavelengths. Each element in the final fingerprint can be quantized into *R* discrete levels depending on the discrete levels of the system. Thus, the theoretical encoding capacity (*C*) is given by

$$C=R^{6n}$$

In this case, with *n* = 10 excitation wavelengths and binary encoding (*R* = 2), the system can generate

$$C=2^{6\left(10\right)}\approx 1.2\times 10^{18}$$

This demonstrates that even with the simplest digitization scheme, our system can theoretically generate over a quintillion unique fingerprints. These unique optical fingerprints highlight the system’s strong encoding capacity and low risk of collision, underscoring its promise for secure identification and anticounterfeiting applications.

### Randomness and Uniqueness Analysis

The optical security codes derived from the binary encoding are presented in Figure 7a, showcasing the final output of the methodology. Each code corresponds to one of the four samples, CIS(530), CIS(530)-PMMA, CIS(650), and CIS(650)-PMMA. The final quantized security codes are arranged side-by-side, enabling a direct visual comparison of their patterns and characteristics. The differences in color distribution and arrangement across the codes reflect the underlying variations in dual-peak emissions, binary encoding, and matrix generation processes. Each security code is visually distinct, reflecting the unique PL emission properties of the corresponding QDs sample. These codes encapsulate the randomness inherent in the dual-peak emissions, translating them into a compact and interpretable format suitable for cryptographic systems. The visual nature of these codes facilitates easy verification and authentication in practical applications, bridging the gap between material-level randomness and digital security.

**Figure 7:**
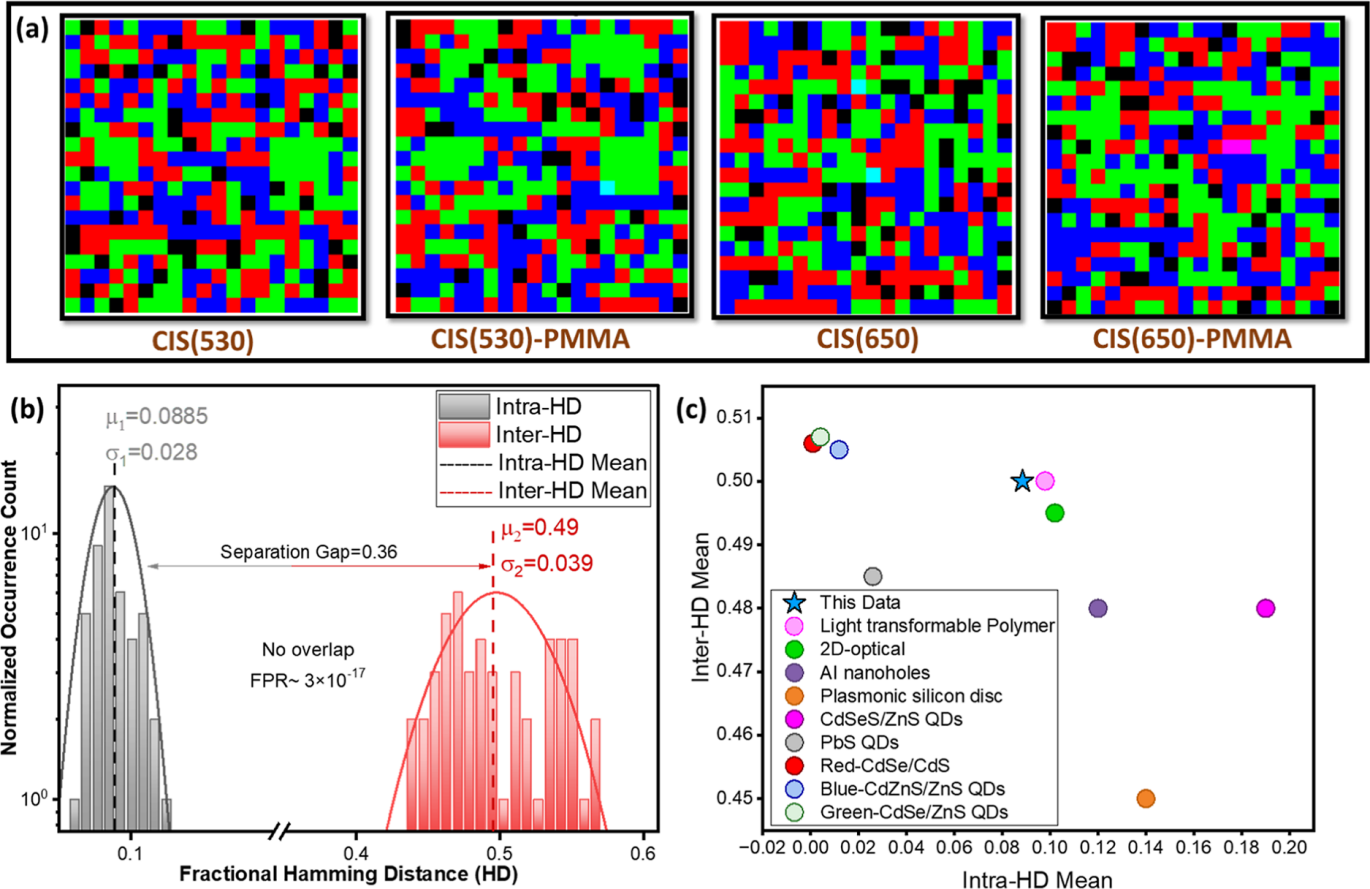
(a) Comparison of the security codes generated from different QDs samples. (b) Histogram of fractional Hamming distances between binary responses, demonstrating the randomness and uniqueness of QD-based security codes. (c) A comparison graph presenting the scale of this work against established optical and nanostructure-based optical PUFs systems.^36,49–55^

To assess the repeatability of our fingerprinting system, we conducted measurements of multiple samples under identical experimental conditions. For each sample, PL emission features were extracted at a specific time for multiple measurements and converted to optical fingerprints. The randomness and uniqueness of the generated binary codes were evaluated by using fractional Hamming distance (HD) analysis. Figure 7b illustrates the distribution of fractional HD for intrasample and intersample comparisons, providing a visual representation of the uniqueness and reproducibility of the binary encoding scheme. The grayish-black bars correspond to intrasample distances, representing the variability within the same sample for multiple consecutive measurements, while the red bars correspond to intersample distances, measuring the distinction between different samples. The distribution shows that intrasample HD are centered around 0.1, indicating that repeated measurements of the same sample generally yield consistent binary responses. This indicates higher reproducibility, ensuring that the same sample produces consistent binary outputs. The reduced variability strengthens the challenge-response consistency, which is a big win in this case. Conversely, the intersample distances remain centered around 0.5, confirming that different QDs samples continue to generate unique binary representations. This level of differentiation is essential for ensuring that no two samples produce similar digital fingerprints, preventing impersonation in authentication systems. The separation between intra- and inter- sample distributions is quite distinct, reducing overlapping. This ensures that intrasample distances do not approach intersample values, thereby enhancing classification accuracy.^48^ It is important to note that this resilience is reflected in our experimental results: despite minor variations in parameters across repeated measurements, the intrasample HD remain clustered around 0.1, confirming that bit assignments are preserved within the tolerance window. This experimentally demonstrated protection ensures that environmental noise does not erode randomness or collapse the mentioned encoding capacity. A well-separated distribution enhances optical PUFs security as it minimizes the likelihood of false positives or misclassification between identical and distinct samples.^26^

To quantitatively assess the theoretically calculated encoding capacity and error probability between different fingerprints, the intra- and inter-sample HD distributions were modeled as independent Gaussian distributions. The intra-HD and inter-HD distributions are characterized by a mean value of μ_1_ and μ_2_ and a standard deviation of σ_1_ and σ_2_, respectively. The key objective is to estimate the probability that two measurements could be incorrectly classified as belonging to the same sample or different samples. To do this, the difference between the two Gaussian distributions is modeled as another Gaussian distribution. This combined distribution reflects, statistically, how well-separated the intra- and inter-HD distributions are. A larger separation gap (Δμ) and smaller combined standard deviation (σ) imply a smaller overlap between the two distributions, leading to lower error probability or false-positive rate (FPR).^48^ The separation between distributions is then normalized to a standard form by calculating the normalized standard deviation and separating the two distributions. The probability that a random intra-HD sample could be misidentified as an inter-HD sample (or vice versa) corresponds to the tail area of the normalized Gaussian distribution beyond the normalized standard deviation. This cumulative tail probability is mathematically described by the complementary error function, denoted erfc. However, since the erfc function accounts for the total probability across both inter- and intra- HD distributions and misidentification could only occur in one direction, the final error probability is computed as

$$P_{error}=\frac{1}{2}\times erfc\left(\frac{\mu_{2}-\mu_{1}}{\sqrt{2}\left(\sqrt{\sigma_{1}^{2}-\sigma_{2}^{2}}\right)}\right)$$

This division by two ensures that only the relevant HD distribution is considered for the misclassification scenario. The resulting *P*_error_ quantifies the overlap between the two distributions: the lower the value is, the less likely the misidentification is to occur. In this study, using the experimental intra-HD and inter-HD distributions (μ_1_ = 0.0885, μ_2_ = 0.49, σ_1_ = 0.028 and σ_2_ = 0.039), the calculated separation gap was 0.36 and the normalized standard deviation value was ≈5.914. Applying the erfc and halving the result, the final experimental probability is estimated to be

$$P_{error}\approx 3\times 10^{-17}$$

This value directly corresponds to the FPR of the system, which is the probability of incorrectly classifying two distinct samples as identical under the applied decision threshold, thus serving as an operational metric for assessing the cloning resistance. The fact that the FPR value is very small indicates that the system can tolerate minor spectral fluctuations without producing bit instabilities. An experimental FPR of 3 × 10^–17^ translates to an effective encoding capacity on the order of 3 × 10^16^ distinguishable fingerprints, underscoring the exceptionally low likelihood of different patterns generating the same encoded output. In practical terms, this means that the encoding capacity remains preserved under real-world conditions, reinforcing the strength of the proposed fingerprinting system. The experimental error probability of our system, when compared to the theoretically calculated one, is still quite apart; yet, we have achieved a very low error probability, which is another confirmation of this system being highly secure.

From an optical PUFs perspective, an ideal system would exhibit minimal intrasample variation (high reliability) and maximal intersample variation (high uniqueness), with a clear separation between the two distributions. The results indicate that the encoding scheme meets these criteria to a reasonable extent, making it a promising candidate for secure authentication applications. Further optimizations, such as refining feature selection or increasing encoding resolution, could help enhance this separation and reduce σ, making this system even stronger against cloning attempts. Furthermore, Figure 7c presents a comparative performance analysis of the proposed system against a range of optical PUFs-based literature studies. The plot maps each method in a two-dimensional space where the *x*-axis represents the mean intra-HD and the *y*-axis denotes the mean inter-HD. This reflects the system’s ability to differentiate between distinct physical instances. The ideal fingerprinting system occupies the upper-left quadrant, i.e., low intra-HD corresponding to high repeatability and high inter-HD leading to high uniqueness. The CIS QDs-based optical fingerprinting system reported in this work is denoted by a star marker in blue color. It is well-positioned in comparison with multiple benchmark systems such as 2D optical encoders, nanohole arrays, lithographically patterned polymers, plasmonic-enhanced surfaces, and multiple QDs.^36,49–55^ These competing systems often rely on either stochastic pattern generation or static topographies to achieve randomness. In contrast, this work achieves repeatability through intrinsic spectral properties, along with the use of structural randomness, while still offering sufficient feature diversity to ensure high intersample variation.

Moreover, Table 1 presents a comparative overview of the optical and nanostructure-based PUF systems, discussed earlier in Figure 7c. While all approaches achieve a balance between uniqueness and reproducibility, their operational principles and readout mechanisms differ significantly. Existing optical PUFs typically rely on multicomponent or hybrid architectures, like color-specific QDs mixtures, plasmonic nanostructures, or polymer-liquid-crystal composites, where the randomness arises from spatial distribution and/or structural variations. Consequently, their optical responses depend strongly on material combinations and fabrication techniques, often requiring complex readout schemes such as speckle imaging, optical scattering, and photocurrent mapping. In contrast, this work’s optical complexity is achieved from a single class of QDs, in which multiple emission peaks are generated intrinsically under variable excitation conditions. This single-material excitation-dependent behavior enables the extraction of diverse spectral features and generates a strong binary fingerprint without introducing external structural randomness. This system clearly exhibits low false-positive rate and a clear separation between intra- and inter- HD distributions, demonstrating reliability and distinctiveness. Our data thus encapsulate the dual strengths of the presented system: it behaves deterministically when it should (intra-HD) and unpredictably when it must (inter-HD). This balance is crucial for real-world applications, where authentication systems must comprehend slight noise or effective drift without sacrificing security. The placement of CIS QDs-based optical fingerprinting in this performance landscape underscores its viability as a competitive and scalable candidate for real-world PUFs applications.

**Table 1:** Comparison of the Scale of This Work against Established Optical and Nanostructure-Based Optical PUFs Systems

Optical Materials	Source of Illumination	Readout Method	Intra-HD Mean	Inter-HD Mean	Bit Error Rate	Bit Uniformity	Reliability/Stability
This Work	Xe lamp (250–345 nm)	PL emission-binary matrix conversion	0.0885	0.5	3 × 10^–17^	∼0.50	>98.7 (no overlap, repeatable PL for days, long-term stability meas.)
PbS QDs^53^	Visible (525 nm)	Photocurrent ranking	0.026	0.485	-	∼0.50	Stable >200 h
Red-CdSe/CdS QDs^54^	540–620 nm (visible)	Fluorescence imaging	0.00102	0.506	FPR ∼ 3.1 × 10^–16^	∼0.499	>90% PL retention
Blue-CdZnS/ZnS QDs^54^	365–450 nm (UV-blue)	Fluorescence imaging	0.01186	0.505	FPR ∼ 2.6 × 10^–16^	∼0.507	>90% PL retention
Green-CdSe/ZnS QDs^54^	450–540 nm (green visible)	Fluorescence Imaging	0.00408	0.507	FPR ∼ 1.1 × 10^–16^	∼0.504	>90% PL retention
CdSeS/ZnS QDs^36^	White light	EPR^a^ spectroscopy	0.15	0.48	Trap density 2 × 10^18^ cm^–3^	-	Reversible photo-EPR^a^ response
PDMS-based light transformable polymer^55^	633 nm (He–Ne) + 470 nm (LED)	Optical speckle-Gabor filter binary	∼0.098	∼0.5	12%	∼0.50	∼100% reversible after 10 cycles
Two-dimensional disordered perforated metallic membrane (Ti)^49^	He–Ne laser 633 nm	Far-field speckle-Gabor filter	∼0.102	∼0.495	<10^–4^	∼0.48	Stable (ΔHD < 0.2 threshold)
Aluminum film with nanoholes^50^	SPR/LSPR^b^ modes (444-653 nm)	Transmission speckle-binary digitization	∼0.1475	∼0.48	No overlap: threshold 0.3	∼0.50	Stable under tilt < 2.5°
Plasmonic silicon disc^51^	100 fs pulse in Si disc	Spectral response-A/D key conversion	∼0.14	∼0.45	No overlap	∼0.50 (passed NIST)	Stable (±50 fs input, ± 50 K temp)

a EPR: Electron Paramagnetic Resonance.

b LSPR: Localized Surface Plasmon Resonance.

### Repeatability and Temporal Stability Analysis

While the HD metrics establish the encoding strength and intersample diversity of the system, it is equally important to demonstrate the temporal consistency of the spectral responses that underpin these digital fingerprints. One of the known challenges with QDs is the decay of their PL emission over time due to factors such as photo-oxidation or environmental degradation,^33^ so we need to test this system over an extended duration to analyze its repeatability and stability. To this end, the repeatability of PL emission characteristics and the resulting binary fingerprints were evaluated over several days using independently prepared samples.

Figure 8a,b shows the PL emission intensity for Peak I and Peak II, respectively, across seven randomly selected CIS(650)-PMMA samples, each measured over three consecutive days for varied excitation wavelengths, but here, for clarity, the fixed excitation at 350 nm is presented. These measurements are performed under identical environmental and instrumental conditions. For each PL measurement at single excitation wavelength, three continuous runs are recorded, and then, the average is plotted here to generate the expected errors at a single point of time. The narrow error bars and consistent intensity levels across the days indicate quite a high degree of spectral stability with minimal photodegradation. Additionally, Figure 8c shifts the focus from raw optical data to complete encoding visualization. A random single CIS(650)-PMMA sample is subjected to PL spectral mapping across a range of excitation wavelengths. From this excitation-dependent dual-peak behavior, six features (per excitation) are extracted after fitting and digitized into a multilevel code (the process is explained earlier in detail). The resulting fingerprint matrices for Day 1, Day 2, and Day 3 are identical in structure, confirming that the full encoding system, from material response to optical fingerprint generation, remains steady over time. This consistency is crucial for any authentication system implemented in uncontrolled scenarios, so the CIS-PMMA system appears resilient to discrepancies due to the passivating effect of the PMMA matrix.^30^

**Figure 8:**
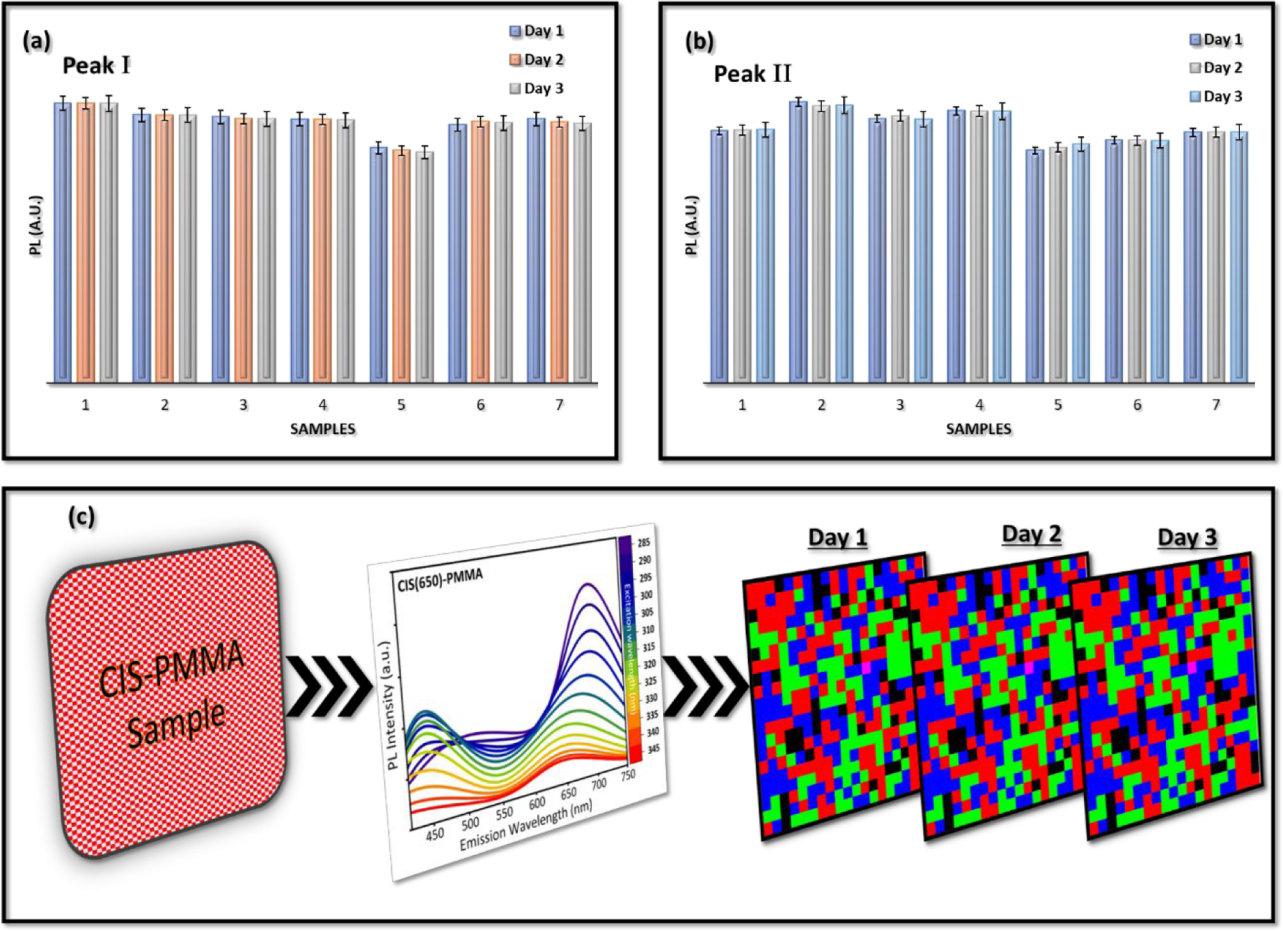
Temporal repeatability measurements for CIS(650)-PMMA samples showing (a,b) PL intensity of Peak I and Peak II, respectively, across three consecutive days for randomly picked CIS(650)-PMMA samples at 350 nm excitation. Error bars represent the standard deviation from three independent measurements per sample. (c) Visual validation of the consistent digital fingerprint generation for day-to-day measurements.

Beyond this short-term repeatability, we further evaluated the long-term PL stability for multiple CIS/ZnS samples in ambient conditions, presented in Figure S9. These samples were evaluated over ∼150 days, and the average PL intensity profiles remained stable. To benchmark stability, three reference checkpoints (τ_1_, τ_2_, τ_3_) were defined at different time intervals. A slight initial decrease in the PL intensity was observed during the first few hours for each sample. This behavior is well-documented for CIS/ZnS QDs and is typically attributed to surface trap equilibration or minor ligand-environmental rearrangements immediately after exposure to ambient conditions.^30^ Once these processes reach equilibrium, the emission stabilizes, and the PL intensity remains essentially constant thereafter, as can be seen in Figure S9. This effect does not alter binary assignments because the drop is uniform across features and well within the predefined tolerance margin. The overall trend shows that intensities do not undergo random degradation but instead remain within a narrow variation range, confirming that QDs-based fingerprints preserve their spectral features over extended durations. The fingerprints generated in our system show both a high encoding capacity and a low FPR value. The digitization process includes a built-in tolerance by defining threshold windows for bit assignment so that small shifts in peak position or intensity remain within the window and do not change the assigned bits. This behavior is consistent with predictive stability assessment frameworks in our earlier work,^56^ where early time measurements (τ_1_, τ_2_) can be used to project an expected value at a later time (τ_3_), and stability is confirmed if the deviation of predicted intensity to actual intensity remains within a tolerance bound. In our case, we directly measure intensities, and the observed variations across τ_1_-τ_3_ remain within the same tolerance range, ensuring that binary assignments are unaffected.

Thus, even over long-term ambient exposure, the CIS QDs demonstrate the bit-level stability required for unclonable fingerprinting. The tolerance threshold feature in the process means that the high encoding capacity is sustainable in real-world deployment. Taken together, these findings validate this fingerprinting platform as both secure and practically applicable, capable of generating and regenerating digital identities without degradation in performance.

## Conclusion

This study establishes a novel fingerprinting methodology based on the consistent dual-peak PL emission of the CIS QDs. By extracting multiple spectral features from each peak under various excitations, this fingerprinting system generates reproducible binary keys with strong uniqueness. Unlike single-peak systems, the additional emission wavelength enhances the encoding capacity without compromising the repeatability. Fingerprints generated from six spectral parameters per excitation wavelength exhibit both high uniqueness and strong temporal fidelity, as verified through HD and the analysis over multiday trials. The dual-peak architecture provides built-in feature diversity, offering a compact yet rich encoding suitable for secure physical identification. Beyond its technical advantages, this approach is notable for its environmental safety, cost-effectiveness, and manufacturing simplicity. Looking ahead, although the current encoding framework has demonstrated good uniqueness and repeatability under controlled laboratory conditions, future work may investigate its resilience under more dynamic environments. This includes assessing the effects of environmental fluctuations, light source instability, or sample aging on fingerprint reliability. The information generated by this dual-peak encoding system could also be utilized strategically to build a strong latent space conditioned on specific QDs properties through deep learning techniques. This approach will enable (i) targeted sampling to generate hypothetical QDs profiles with optimized emission properties and (ii) rapid fingerprint validation for real-time authentication in security applications.

These results advance the field of optical PUFs by introducing a system that is spectrally rich, fabrication-friendly, and digitally strong. This approach can be extended to mobile authentication systems and dynamic excitation-based keys, thereby bridging the gap between lab-scale photonic tags and real-world security deployment.

## Experimental Section

### Materials and Chemicals

CuInS_2_/ZnS QDs with nominal emission wavelengths of 530 and 650 nm were purchased from NN-Crystals US Corporation. According to the supplier, the average core diameter for CIS(530) QDs was 2.0 ± 0.5 nm, and for CIS(650) QDs, it was 3.5 ± 0.5 nm, with shell thicknesses of approximately 2.0 and 2.5 nm, respectively. The size variation (±values) reflects the manufacturer-reported particle size distribution. The stabilizing ligand used for these QDs was oleic acid/oleylamine.

Poly­(methyl methacrylate) (PMMA), purchased from Sigma-Aldrich, USA, served as the polymer matrix to enhance QD stability and facilitate the integration of QDs to be deposited over the substrate. All of the chemicals and materials used in this study were procured from commercial suppliers and used without further purification. All samples were deposited onto clear glass substrates.

### Sample Preparation

Four distinct samples were prepared for PL analysis. The first two samples consisted of CIS QDs emitting at 530 and 650 nm, without PMMA. The dispersion of these samples was in the ratio of ∼2 mg/mL QDs in the solvent. The other two samples were prepared by embedding the QDs emitting at 530 and 650 nm into a PMMA matrix. Following that, the same dispersion of QDs was dropwise added to this precursor solution to form the QD-PMMA matrix. The QDs were dispersed from their raw form without any additional purification steps. So, the samples we have are

a CIS QDs emitting at 530 nm without PMMA (CIS(530))
b CIS QDs emitting at 530 nm embedded in PMMA (CIS(530)-PMMA)
c CIS QDs emitting at 650 nm without PMMA (CIS(650))
d CIS QDs emitting at 650 nm embedded in PMMA (CIS(650)-PMMA)

The samples were spin-coated onto glass substrates to ensure uniform film formation to study and compare the PL measurements. The spin-coating conditions were carefully controlled, with a spin speed of 3500 rpm for 8 s, followed by drying at room temperature for half an hour for the evaporation of residual solvent. Furthermore, after drying, the film was encapsulated with an additional PMMA overlayer.

## Characterization

The film’s thickness and roughness were analyzed by tapping mode AFM using a Park XE7 system. The steady-state PL spectra were recorded using a FLS1000 photoluminescence spectrometer (Edinburgh Instruments). The excitation wavelengths ranged from 250 to 345 nm, for all samples, with excitation provided by a Xe lamp in conjunction with a monochromator. This system automatically inserts appropriate long-pass filters to block excitation scatter and Raman contributions, ensuring an accurate PL emission signal collection.

TRPL measurements were also performed using the same FLS1000 spectrometer equipped with a time-correlated single-photon counting module. A pulsed diode laser (λ = 375 nm) was used as the excitation source, with a pulse duration of <100 ps and a repetition rate of 1 MHz. The system’s temporal resolution was ∼150 ps and the instrument response function (IRF) reconvolution was enabled during measurements, no separate IRF traces were acquired, and the software’s IRF kernel was used during fitting. Emission decays were collected at the respective emission maxima of the two peaks for each sample. For CIS(530)-PMMA, TRPL was recorded at ∼530 nm (Peak I) and ∼450 nm (Peak II), while for CIS(650)-PMMA, the decays were measured at ∼650 nm (Peak I) and ∼500 nm (Peak II). This allowed separate evaluation of recombination dynamics associated with the long-wavelength (core-related) and short-wavelength (interfacial/shell-related) emissions.

The PL decay curves were fitted using a multiexponential decay function of the form

$$I\left(t\right)=\sum_{i}B_{i}e^{-t/\tau_{i}}+y_{o}$$

where *B*_*i*_ and τ_*i*_ are the pre-exponential factor and lifetime of the *i*th component, respectively, and *y*_o_ represents the baseline offset accounting for background counts, which was included in the fitting to ensure accurate estimation of the decay parameters. The fractional contribution of each component was calculated as

$$f_{i}=\frac{B_{i}}{\sum B_{i}}$$

and the average lifetime was determined as

$$\tau_{avg}=\sum_{i}f_{i}\tau_{i}$$

This approach captures the weighted average decay time corresponding to the measured emission band. Fitting was performed in Fluoracle software with χ^2^ values close to ∼1.1, confirming a good fit quality.

### Fitting Method

A Gaussian fitting was employed to isolate and quantify the emission peaks, extracting critical parameters such as emission wavelength, intensity, and FWHM. All spectra were first baseline-corrected to remove background contributions, and the same fitting procedure was applied identically to all data sets for both CIS(530) and CIS(650) across all excitation wavelengths. Measurements were acquired under identical spectrometer settings to ensure comparability. The Python code was used to process the data of the samples under the same environment and with the same algorithm. The code uses a skewed Gaussian equation to fit a single peak from the raw data PL spectra and a double-skewed Gaussian equation for the double peaks in PL spectra.

1 $$f\left(x\right)=a\;\exp\left(-\frac{{\left(x-\mu \right)}^{2}}{2\sigma^{2}}\right)\left(1+\mathrm{erf}\left(\frac{\lambda \left(x-\mu \right)}{\sqrt{2}\sigma }\right)\right)$$

2 $$f\left(x\right)=a_{1}\;\exp\left(-\frac{{\left(x-\mu_{1}\right)}^{2}}{2\sigma_{1}^{2}}\right)\left(1+\mathrm{erf}\left(\frac{\lambda_{1}\left(x-\mu_{1}\right)}{\sqrt{2}\sigma_{1}}\right)\right)+a_{2}\;\exp\left(-\frac{{\left(x-\mu_{2}\right)}^{2}}{2\sigma_{2}^{2}}\right)\left(1+\mathrm{erf}\left(\frac{\lambda_{2}\left(x-\mu_{2}\right)}{\sqrt{2}\sigma_{2}}\right)\right)$$

Equations 1 and 2 show the fitting function, where the parameter *a* is the amplitude, which reflects the height of the peaks, μ is the peak wavelength or the center wavelength of the peak, and σ is the standard deviation, which is related to FWHM.

3 $$FWHM=2\sigma \cdot \sqrt{2\;\ln\left[a_{1}\;\exp\left(-\frac{{\left(x-\mu_{1}\right)}^{2}}{2\sigma_{1}^{2}}\right)\left(1+\mathrm{erf}\left(\frac{\lambda_{1}\left(x-\mu_{1}\right)}{\sqrt{2}\sigma_{1}}\right)\right)+a_{2}\;\exp\left(-\frac{{\left(x-\mu_{2}\right)}^{2}}{2\sigma_{2}^{2}}\right)\left(1+\mathrm{erf}\left(\frac{\lambda_{2}\left(x-\mu_{2}\right)}{\sqrt{2}\sigma_{2}}\right)\right)\right]}$$

Equation 3 shows the calculation of the FWHM, λ is the skewness parameter, which controls the skewness of each peak, and *x* is an independent variable, which represents the actual wavelength.

To fit the spectra, first, the skewed Gaussian function and double skewed Gaussian function were defined. Then, the FWHM was defined using σ. Before fitting, the initial parameters of the fitting function were estimated and given as an input. Also, the parameters were given boundaries to make sure that all of them were within a reasonable range. During the fitting, the least-squares method was used to search for the closest result to the original values, and the resulting parameters were stored in Excel files.

### Data Encryption

The PL parameters obtained after fitting were processed by using Python, where they were normalized to a consistent scale and digitized through a thresholding algorithm. The binary encoding of each parameter ensured that material-specific randomness was translated into a machine-readable format.

## Digitization of Optical Fingerprint

Custom-developed MATLAB (R2023a) and Python (v3.12.3) scripts were used for binary digitization, matrix transformations, and quantization-based fingerprint generation. This hybrid coding framework allows full control over each step of the spectral-to-digital conversion process. The resulting 2D composite output/display was further processed through a quantization algorithm implemented in Python, where pixel values from each colored matrix were discretized into predefined intensity levels. This quantization step converted the continuous spectral features into a finite set of encoding states, effectively increasing the strength and minimizing minor fluctuations during the measurements. By constraining pixel intensities to controlled levels, the quantized display functions as a noise-tolerant, high-capacity optical key that can be consistently regenerated under repeated measurements. The final output, a quantized digital optical fingerprint, serves as a visually interpretable yet algorithmically secure representation of the sample’s spectral identity, optimized for cryptographic integration and authentication applications.

## Supplementary Material



## Notes

AFM analysis, photoluminescence spectra at different excitation wavelengths for CdSe/ZnS, representative Gaussian fitting examples, extracted PL parameters as a function of excitation wavelength, binary encoding, long-term stability, and fingerprinting of additional samples (PDF)
