# Supplementary material for: High-Capacity Optical Fingerprinting Using Dual-Peak Photoluminescence of Quantum Dots
Source: ACS Appl Mater Interfaces. 2025 Dec 22;18(1):3086–101. doi: 10.1021/acsami.5c19508 (PMC12781110; doi:10.1021/acsami.5c19508)
Supplement: Supplementary file 1 [file am5c19508_si_001.pdf]

Supporting Information

High-Capacity Optical Fingerprinting using Dual-Peak Photoluminescence of Quantum Dots

Syeda Ramsha Ali[*,a], *Stephen V. Kershaw*[b], *Yinglong Zhu*[c], *Ahmed A.Z. Dawoud*[d], *Yueyu Guo*[a], *Kees De Groot*[a], *Nema M. Abdelazim*[**,a]

[a] *School of Electronics and Computer Science, University of Southampton, Southampton SO17 1BJ, United Kingdom.*

[b] *Department of Physics and Materials Science, City University of Hong Kong, Hong Kong S.A.R*

[c] *Microelectronics Thrust, The Hong Kong University of Science and Technology (Guangzhou), Guangzhou, 511455, China.*

[d] *School of Biological Sciences, University of Southampton, Southampton SO17 1BJ, United Kingdom*

[*] sra1r23@soton.ac.uk [**] nema.abdelazim@soton.ac.uk

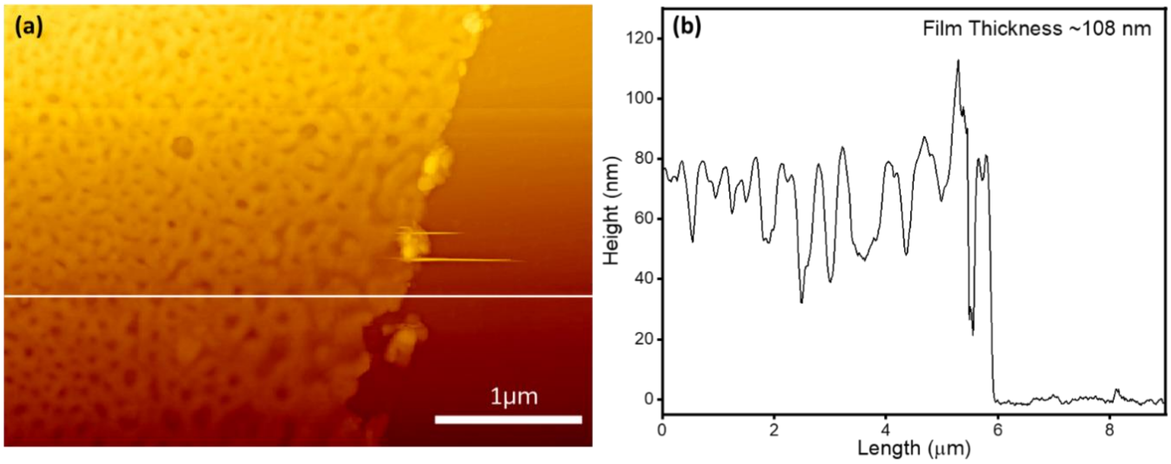

Figure S1: (a) AFM topography of the deposited film over a 4 μm × 4 μm scan area with the **(b)** extracted height profile, indicating a non-uniform surface and a step height of approximately ~108 nm corresponding to the film thickness.

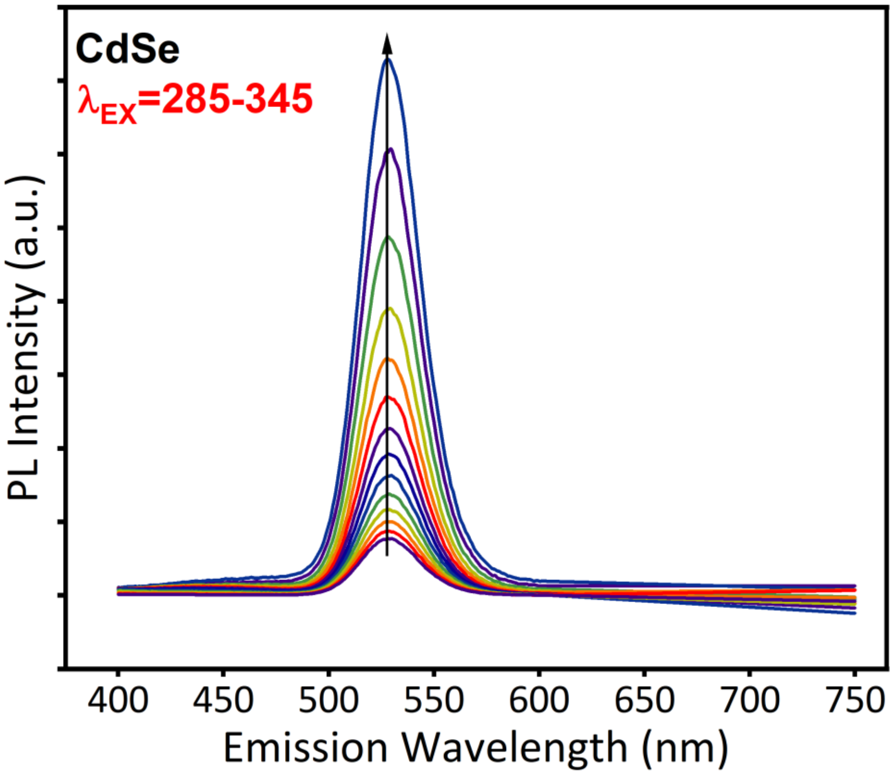

Figure S2: Full PL spectra of CdSe film recorded under a range of excitation wavelengths from 285 nm to 345 nm.

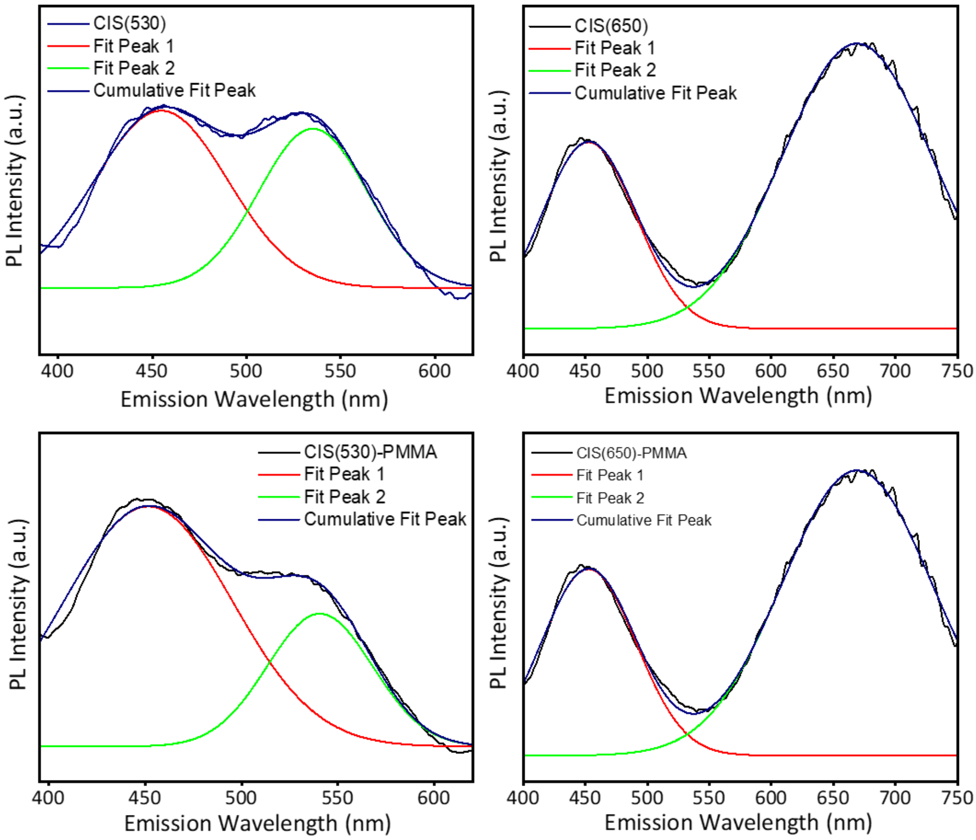

Figure S3: Representative two-Gaussian peak fitting of the PL emission spectrum for all the samples. The experimental data (black line) is overlaid with the fitted Gaussian components (coloured curves) and cumulative fit (blue line), demonstrating accurate dual-peak emissions.

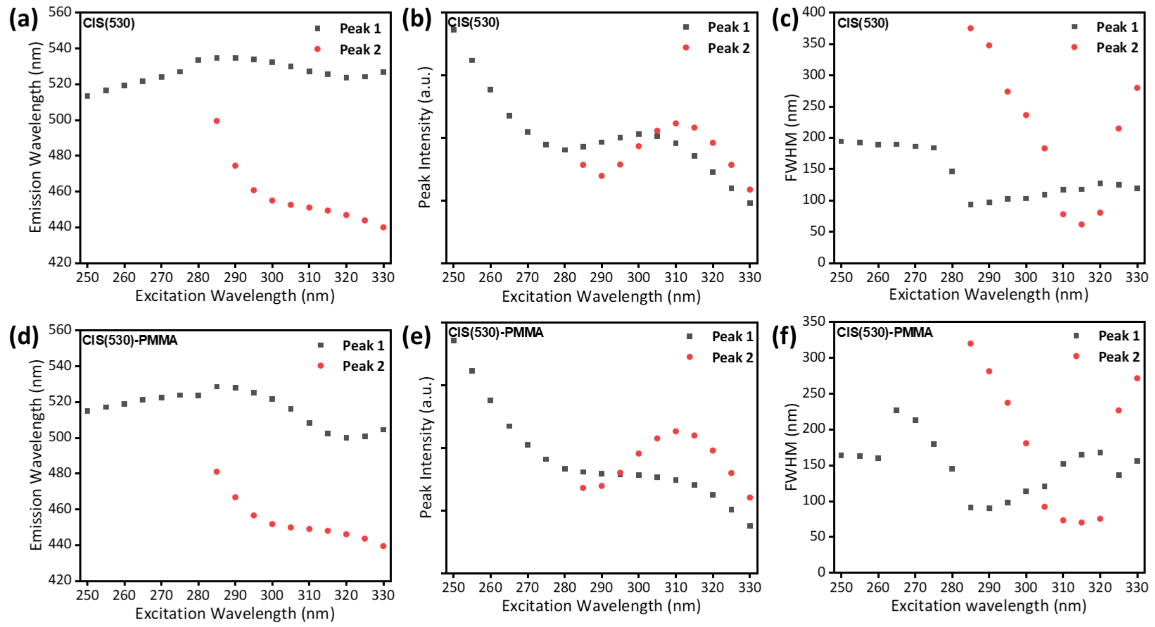

Figure S4: Extracted PL parameters as a function of excitation wavelength for CIS(530) and CIS(530)-PMMA composites: **(a-c)** show the evolution of emission wavelength, peak intensity, and FWHM for Peak 1 (grey squares) and Peak 2 (red dots) in CIS(530), **(d–f)** display the same for CIS(530)-PMMA.

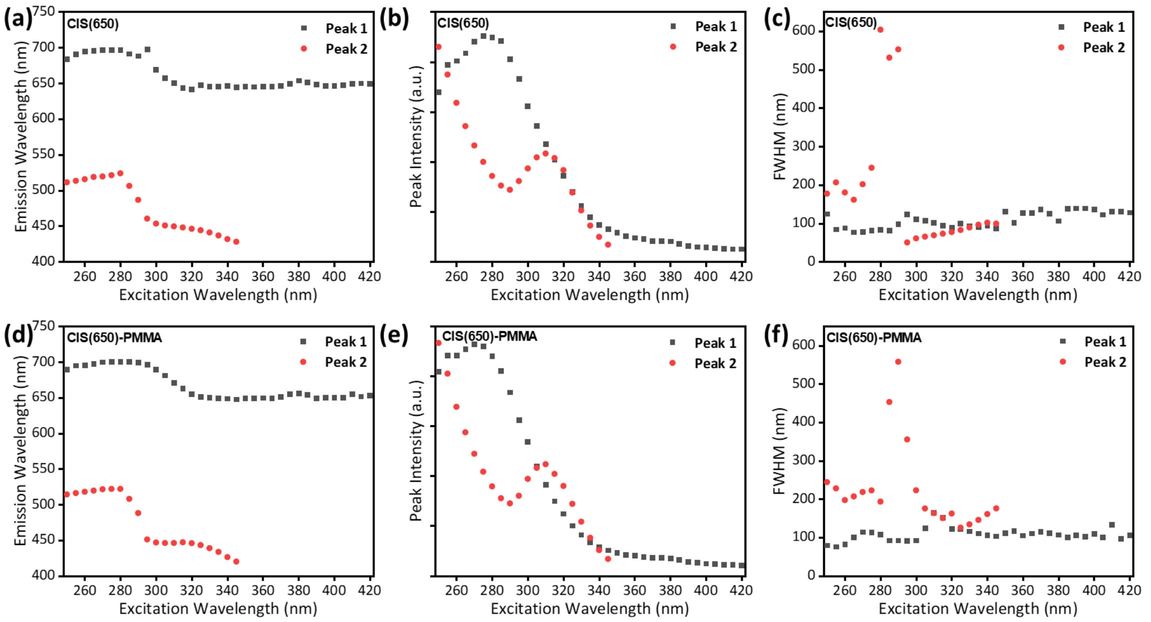

Figure S5: Extracted PL parameters as a function of excitation wavelength for CIS(650) and CIS(650)-PMMA composites: **(a-c)** show the evolution of emission wavelength, peak intensity, and FWHM for Peak 1 (grey squares) and Peak 2 (red dots) in CIS(530), **(d–f)** display the same for CIS(650)-PMMA.

Table S1: Binary encoding of PL parameters for QD samples

Sample ID			***Binary Codes***
CIS(530)	Peak 1	*Emission Wavelength*	1111100000
		PL Intensity	1111111000
		FWHM	0000011111
	Peak 2	*Emission Wavelength*	1100000001
		PL Intensity	0001111100
		FWHM	1111000001
CIS(530)-PMMA	Peak 1	*Emission Wavelength*	1111100010
		PL Intensity	1111111100
		FWHM	0001011111
	Peak 2	*Emission Wavelength*	1100000110
		PL Intensity	0001111000
		FWHM	1110000011
CIS(650)	Peak 1	*Emission Wavelength*	1110010000
		PL Intensity	1110100000
		FWHM	0011110000
	Peak 2	*Emission Wavelength*	1100000000

		PL Intensity	0000000010
		FWHM	1100100001
CIS-PMMA	Peak 1	*Emission Wavelength*	1011100000
		PL Intensity	1111000000
		FWHM	0000111110
	Peak 2	*Emission Wavelength*	1100000100
		PL Intensity	0000100000
		FWHM	1110000000

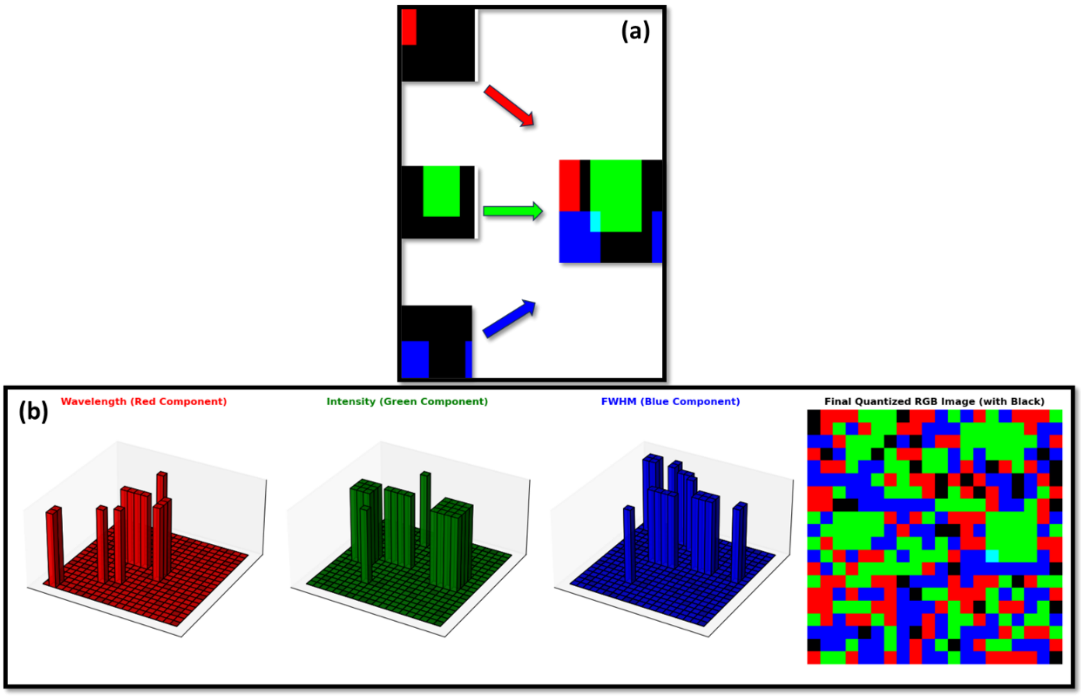

Figure S6: A visual representation of the process used to generate a quantized security code by encoding spectral parameters into a structured colour-mapped format for CIS(530)-PMMA **(a)** the transformation of binary spectral data into individual colour components and **(b)** the corresponding 3D quantization of these components, leading to the final quantized optical security image

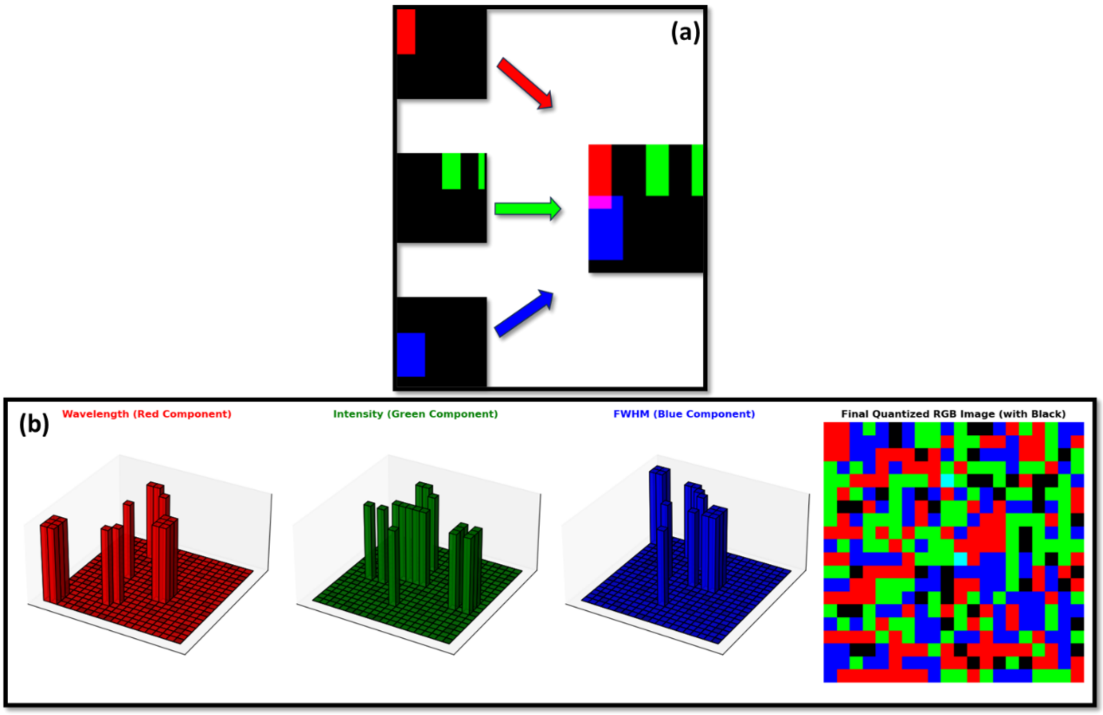

Figure S7: A visual representation of the process used to generate a quantized security code by encoding spectral parameters into a structured colour-mapped format for CIS(650) **(a)** the transformation of binary spectral data into individual colour components and **(b)** the corresponding 3D quantization of these components, leading to the final quantized optical security image

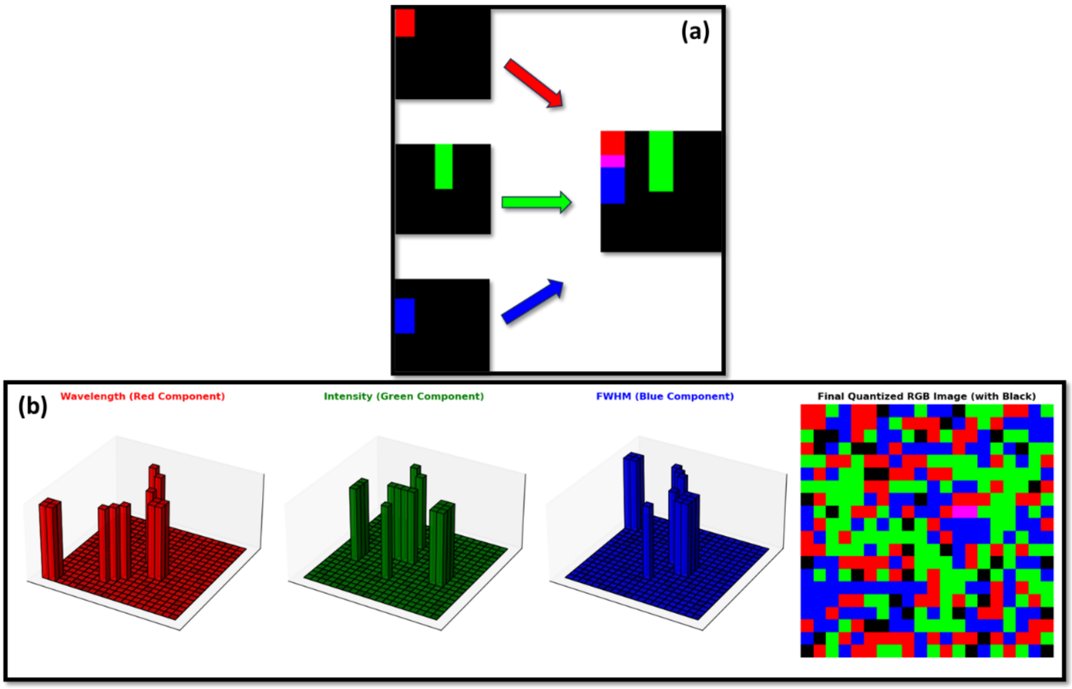

Figure S8: A visual representation of the process used to generate a quantized security code by encoding spectral parameters into a structured colour-mapped format for CIS(650)-PMMA **(a)** the transformation of binary spectral data into individual colour components and **(b)** the

corresponding 3D quantization of these components, leading to the final quantized optical security image

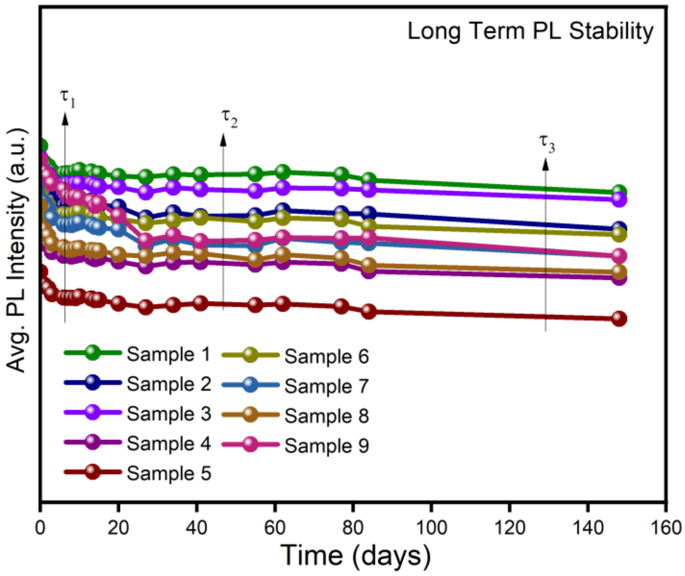

Figure S9: Long-term PL intensity stability of multiple CIS/ZnS samples monitored under open environment.
